# Focality of the Induced E-Field Is a Contributing Factor in the Choice of TMS Parameters: Evidence from a 3D Computational Model of the Human Brain

**DOI:** 10.3390/brainsci10121010

**Published:** 2020-12-18

**Authors:** Deepika Konakanchi, Amy L. de Jongh Curry, Robert S. Waters, Shalini Narayana

**Affiliations:** 1 Biomedical Engineering, University of Memphis, Memphis, TN 38152, USA; adejongh@memphis.edu (A.L.d.J.C.); rwaters@uthsc.edu (R.S.W.); 2 Department of Orthopaedic Surgery and Biomedical Engineering, University of Tennessee Health Science Center, Memphis, TN 38163, USA; 3 Department of Anatomy and Neurobiology, University of Tennessee Health Science Center, Memphis, TN 38163, USA; 4 Department of Pediatrics, University of Tennessee Health Science Center, Memphis, TN 38163, USA; 5 Neuroscience Institute, Le Bonheur Children’s Hospital, Memphis, TN 38163, USA

**Keywords:** focality, TMS, transcranial magnetic stimulation, FEM, TMS optimization, coil orientation, volume of activation

## Abstract

Transcranial magnetic stimulation (TMS) is a promising, non-invasive approach in the diagnosis and treatment of several neurological conditions. However, the specific results in the cortex of the magnitude and spatial distribution of the secondary electrical field (E-field) resulting from TMS at different stimulation sites/orientations and varied TMS parameters are not clearly understood. The objective of this study is to identify the impact of TMS stimulation site and coil orientation on the induced E-field, including spatial distribution and the volume of activation in the cortex across brain areas, and hence demonstrate the need for customized optimization, using a three-dimensional finite element model (FEM). A considerable difference was noted in E-field values and distribution at different brain areas. We observed that the volume of activated cortex varied from 3000 to 7000 mm^3^ between the selected nine clinically relevant coil locations. Coil orientation also changed the induced E-field by a maximum of 10%, and we noted the least optimal values at the standard coil orientation pointing to the nose. The volume of gray matter activated varied by 10% on average between stimulation sites in homologous brain areas in the two hemispheres of the brain. This FEM simulation model clearly demonstrates the importance of TMS parameters for optimal results in clinically relevant brain areas. The results show that TMS parameters cannot be interchangeably used between individuals, hemispheres, and brain areas. The focality of the TMS induced E-field along with its optimal magnitude should be considered as critical TMS parameters that should be individually optimized.

## 1. Introduction

Transcranial magnetic stimulation (TMS) is used to study brain function by applying localized magnetic fields in a noninvasive manner. The diagnostic and therapeutic applications of TMS have expanded over the last decade, in part due to its non-invasive nature and excellent safety record [1]. Currently, TMS has been approved by the US Food and Drug Administration (FDA) for use in presurgical mapping of eloquent cortex and for treating major depression, chronic pain, migraine, and obsessive compulsive disorder [2,3]. The behavioral effects of TMS are thought to be broadly mediated via the secondary electrical field (E-field) induced in the underlying cortex by the primary current flowing in the TMS coil. However, the exact mechanisms of interaction of TMS with the neuronal elements are yet to be fully characterized.

An important prerequisite for examining the interactions between TMS and cortex is to know the magnitude and the spatial distribution of the induced E-field at the site of stimulation. The E-field magnitude and its distribution are influenced by TMS parameters such as the type of coil, its orientation, rate, and intensity and individual parameters such as the columnar organization and the cortical folding pattern at the site of stimulation and interactions between the two types of parameters. These factors have to be taken into consideration to accurately estimate the magnitude and the spatial distribution of the induced E-field [4,5,6,7]. Computational models provide an excellent platform to calculate the E-field magnitude and distribution based on different choices of TMS parameters while also taking into account their interaction with individual cortical anatomy [6,8,9,10,11,12]. Over the last two decades, computation modeling studies have attempted to examine the effects of coil type [13], orientation [14,15,16,17,18], and the rate and intensity [8,19,20,21] and site of stimulation [16,22,23] on the induced E-field in the underlying neuronal tissues using different finite element method (FEM) platforms. To the best of our knowledge, no prior studies evaluate the volume of activated cortex in correlation to different TMS parameters and determine any relationship between the volume of activation and optimal TMS induced E-field.

Among the different computational models available, a 3D FEM based pipeline for individualized head modeling, tissue segmentation, and E-field modeling called SimNIBS (www.simnibs.org) has gained popularity because of its comprehensive approach and ease of use. Additionally, the maximal E-field estimated by SimNIBS in specific areas of the motor cortex based on coil orientation was validated with experimental measurements [19]. The SimNIBS-predicted orientation/location combinations were found to more effectively stimulate the cortical site and produce motor evoked potentials when compared to other orientation/location combinations estimated by other models [24]. Recently, Aberra and colleagues integrated the neuronal components into a 3D FEM model in SimNIBS to elucidate the nature of TMS interaction with different neural elements and the influence of TMS parameters on the neural response [8]. These previous researchers demonstrate that SimNIBS is a powerful tool to accurately model the E-fields generated by TMS and is well suited to examine the effects of different TMS parameters in a comprehensive and systematic way.

In the present study, we wanted to identify which of the TMS parameters can be generalized across brain areas and individuals and which parameters need to be optimized on an individual basis. To answer this question, we examined two TMS parameters (intensity and orientation) and one individual-based parameter (site of stimulation) on the magnitude and spatial distribution of the E-field using a standardized normal brain template, the Montreal Neurological Institute (MNI) atlas. We selected clinically relevant cortical locations in both hemispheres covering the primary motor, somatosensory, auditory, and visual cortices, inferior frontal gyrus, dorsolateral prefrontal frontal cortex, and auditory association areas. First, we examined whether the E-field strength and distribution in the underlying cortex and the volume of activated cortex differed between homologous positions in the two hemispheres and in the same hemisphere at different locations. We then examined, for a given location, the impact of different intensities and coil orientations on the strength, distribution of the E-field, and volume of activation. We tested the hypothesis that the underlying cortical geometry would influence the induced E-fields generated by a given intensity and orientation. Therefore, we expected to find both the strength and extent of the E-field induced in homotopic areas with similar cortical geometry to be similar for a given TMS intensity and orientation whereas the induced E-fields for a given intensity and coil orientation would be more variable across different brain regions in the same hemisphere that have different cortical geometry. Based on the results of the induced E-fields and volume of activated cortex, we wanted to determine if any relationship exists between the two data sets for different coil locations and orientations.

## 2. Methods

### 2.1. E-Field Modeling

The E-field induced by TMS was computed in a realistic, 3D volume conductor model of the human brain using the open-source simulation package SimNIBS v3.1.2 [25]. The finite element head model used in this study is included in the SimNIBS example dataset and reconstructed by applying the headreco pipeline [26] to the 1 mm MNI T1 template. The five tissue types included in the model are white matter (WM), gray matter (GM), cerebrospinal fluid (CSF), bone, and scalp [27]. Isotropic tissue conductivities of 0.126 Siemens/meter (S/m) for WM, 0.275 S/m for GM, 1.654 S/m for CSF, 0.010 S/m for bone, and 0.465 S/m for scalp were assigned based on previous studies [28]. The built-in SimNIBS model of the Magstim 70 mm figure-of-8 coil generating a monophasic pulse was chosen as the TMS coil for all simulations [29,30]. The quasistatic FEM [16,31] is used in SimNIBS, where a linear system of equations of the form Mu = b are solved to compute the induced E-field. M is a large, sparse stiffness matrix, u is an array of nodal electric potentials, and b includes boundary conditions and source parameters as described by Saturnino et al. [32]. As modeled in SimNIBS, the E-field input is in the form of dI/dt in units of A/µs. TMS simulation begins by calculating the change in magnetic vector potential, i.e., dA/dt, in the elements of the volume conductor mesh for the given coil type, position, and E-field input. Finally, FEM calculation of the maximum E-field at the cortical surface is expressed as the norm or the magnitude of the E-field in units of volts/meter (V/m) [32]. SimNIBS only visualizes the magnitude, i.e., vector length of the E-field, and the vector is not further decomposed into normal and tangential components [32]. The model also outputs volume of gray matter that is exposed to an E-field greater or equal to 50% and 75% of the peak value as an index of focality of TMS. In this manuscript, we reported E-field greater or equal to 75% of the peak value, which is a reasonable representation of the volume of cortex effectively stimulated/activated by TMS [32]. It is expected that the targeted cortex should be exposed to at least 75% of the maximum E-field in order for TMS to be effective especially in the context of therapeutic applications of TMS. For example, in depression treatment where TMS is applied at 120% of the motor threshold, 75% of the maximum E-field falls below the threshold, and therefore, realistically, cortex exposed to subthreshold E-field magnitudes do not receive the therapeutic dose of E-field.

### 2.2. Model Verification

**Figure 1:**
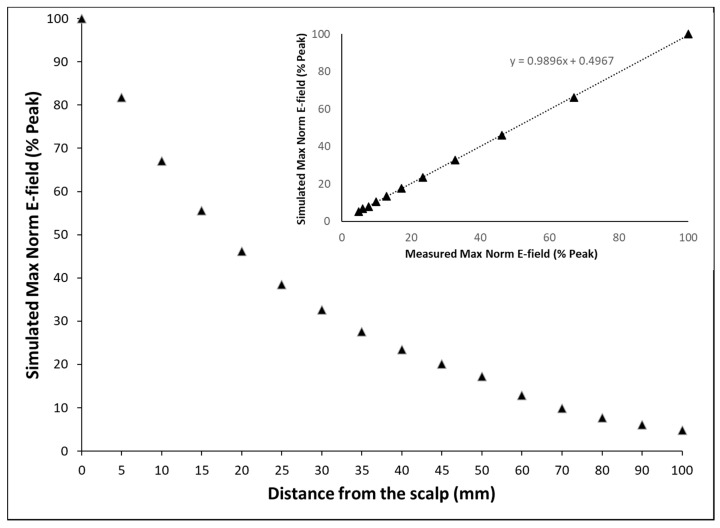
Decay of induced E-field over distance as modeled by SimNIBS. The simulated max norm E-field is expressed as a percent of maximum norm E-field at a certain distance away from scalp to that at the scalp surface. The E-field decays by the square of the distance of the coil from the scalp; inset: normalized E-field as modeled by SimNIBS vs. measured E-field expressed as % E-field at the surface of the coil. Measured max norm E-field is expressed as the ratio of the maximum norm E-field at a certain distance in air to that at the coil surface. The simulation shows excellent agreement with the measured data with a R^2^ = 0.98.

We verified the Magstim 70 mm figure-of-8 coil E-field model used in SimNIBS against the measured values. The Magstim coil E-field was measured using a custom-designed 3D eddy current probe connected to a digital oscilloscope [10]. The probe was moved from the coil surface (0 mm) to 100 mm from the coil in air, in the absence of a physical head and E-field measurements were made at the site of maximal E-field, referred to as the hot spot [10]. The maximum E-fields measured at different distances were expressed as a percent of E-field at the surface of the coil. In the SimNIBS simulation model, the same E-field decay was simulated by moving the TMS coil from the scalp (mimicking measurement at the coil surface, 0 mm distance) to 100 mm away from the scalp (analogous to measurement at 100 mm from the coil surface in air) in 5 mm steps. The maximum norm E-field calculated at the cortical surface was recorded for each location of the coil at different distances from the scalp. As with the measured E-field, the E-fields modeled at different distances from the scalp were expressed as a percent of E-field at the scalp surface. Figure 1 shows that in the simulation model, the induced E-field decays by the square of the distance of the coil from the scalp and is in excellent agreement with measured values, with an R^2^ of 0.98.

### 2.3. Choice of Brain Areas

**Figure 2:**
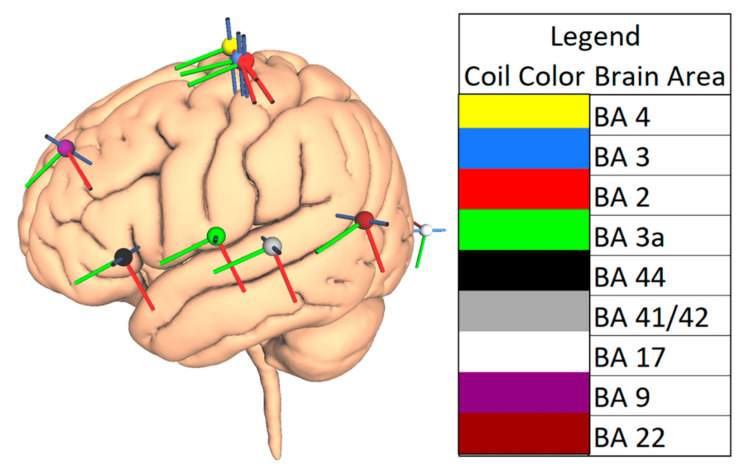
Cortical brain areas studied. The positions of the nine clinically important coil locations (projected on the cortical surface) selected for this study are shown. For example, BA4 represents Brodmann area 4, which is representative of the primary motor cortex. The legend lists the color codes and names of coil locations.

Nine cortical regions were selected to represent four lobes (frontal, temporal, parietal, and occipital) in each hemisphere. The regions were selected based on their clinical importance as treatment targets and in the context of presurgical mapping. These regions also differed in their gyral folding patterns. The cortical regions examined in this study were the primary motor cortex (Brodmann area (BA) 4), primary somatosensory cortex (BA 3, BA2, and BA 3a), inferior frontal gyrus (BA 44), including Broca’s area in the left hemisphere, primary auditory cortex (BA 41/42), primary visual cortex (BA 17), dorsolateral prefrontal cortex (BA 9), and the auditory association cortex (BA 22), including Wernicke’s area in the left hemisphere. Figure 2 displays the positions of all selected coil positions.

### 2.4. TMS Parameters

First, we used the SimNIBS platform to model the E-field induced by a TMS intensity of 150 A/µs and coil oriented towards the nose at each of the cortical locations and extract the maximum E-field and the volume of cortex exposed to at least 75% of the peak E-field for each coil location. Then, we examined the effect of different TMS intensities and orientations on the modeled maximum E-field and the volume of cortex exposed to at least 75% of the peak E-field for these brain areas. We examined three intensity inputs (dI/dt): 100, 150, and 200 A/µs, emulating different machine outputs. Although there is no uniformity across different TMS machines with respect to the stimulator output, based on measured E-field data from a Magstim TMS system (Magstim^®^ 200²), we estimated that an input dI/dt of 100 A/µs to be an approximation of 50% machine output; dI/dt of 150 A/µs to be 75% and dI/dt of 200 A/µs to be 100% machine output.

**Figure 3:**
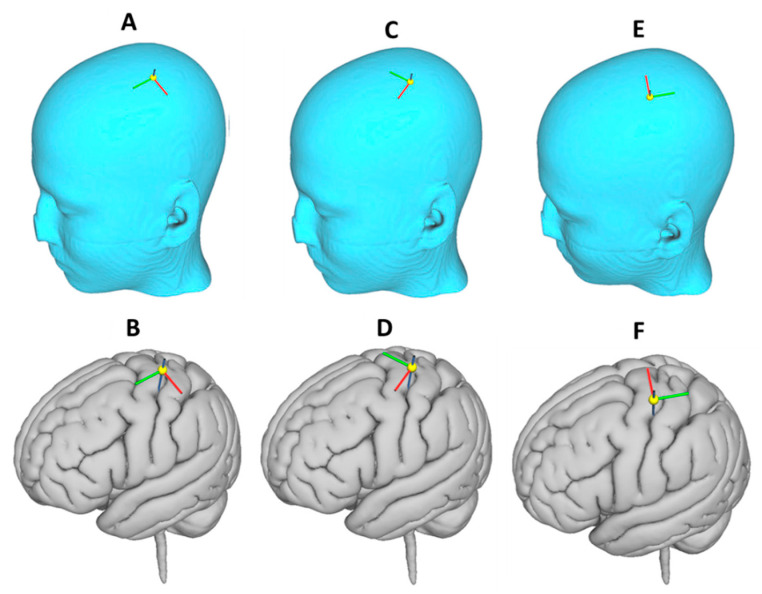
Transcranial magnetic stimulation (TMS) coil orientations examined. The green limb indicates the direction of current in the coil. (**A**,**B**) Current flow towards Nz. (**C**,**D**) Current flow towards FCz. (**E**,**F**) Current flow towards Oz. Nz, FCz, and Oz are standard positions in the 10-10 electrode system for EEG recording. The (**A**,**C**,**E**) panels show the coil orientation on the skull and the (**B**,**D**,**F**) panels show the coil projection on the cortical surface.

Finally, we examined the effect of changing the orientation of the current flow in the coil. The most commonly used coil orientation in clinical studies is the coil pointing to the nose, with the current flowing in antero-posterior direction (see Figure 3A), parallel to the line connecting external auditory meatus and nasion [33,34,35]. This coil orientation was modeled in SimNIBS as current flow pointing towards the Nz electrode position in the 10-10 electrode system for electroencephalogram (EEG) recording [36] (see Figure 3A,B). Following this, simulations were performed after rotating the coil by approximately 60°, with current flow directed towards the vertex (FCz electrode position in the 10-10 EEG system, Figure 3C,D) and then by approximately 180°, with current flow directed towards the occipital pole (Oz electrode position in the 10-10 EEG system, Figure 3E,F). The modeled maximum E-field and the volume of the cortex exposed to at least 75% of the peak E-field were reported for each combination of the cortical location and orientation.

## 3. Results

### 3.1. Maximum E-Field at Different Cortical Locations

**Figure 4:**
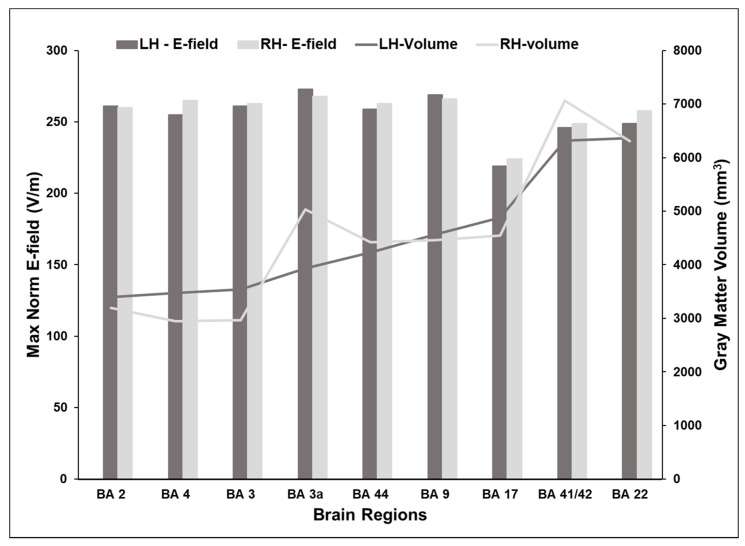
Maximum norm E-field and volume of activated cortex in homologous locations in the left and right hemispheres with the coil oriented towards the nose (EEG position: Nz). The TMS intensity was set at dI/dt = 150 A/µs. The maximum norm or strength of E-fields are shown as bars on the y-axis on the left. Very small differences in norm E-fields were observed between the homologous locations in the left and right hemispheres. The volume of cortex exposed to at least 75% of maximum E-field is plotted as lines on the y-axis on the right. The volume of activated cortex differed at homologous locations in the left and right hemispheres especially in BA 3, BA 3a, and BA 41/42. LH—left hemisphere; RH—right hemisphere.

We executed FEM simulations for each of the nine selected cortical locations in the two hemispheres with the TMS intensity set at 150 A/µs and coil oriented towards Nz for which the maximum E-field values are displayed in Figure 4. Table 1 lists the E-field values for the different intensities of 100, 150, and 200 A/µs for both coil orientations Nz (nose) and FCz (vertex). On average, there was a very small difference (2% average, range 0–4%) between the maximum E-fields at homologous locations in the left and right hemispheres. However, within the same hemisphere, the maximum E-field at the cortical surface was found to differ considerably between brain areas. The highest E-fields were induced in the primary somatosensory (BA 3a) and the dorsolateral prefrontal (BA 9) cortices. However, for the same intensity and orientation of TMS, the maximum E-fields in the visual cortices (BA 17) were lower by 18%. E-fields induced in the primary auditory (BA 41/42) and association auditory (BA 22) cortices were nearly 10% less than E-fields noted at BA 3a and BA 9. In the remaining brain areas, there was approximately a 4% difference in the maximum E-fields when compared to BA 3a and BA 9. Since the homologous areas were found to have comparable E-fields, further comparisons for each cortical location were made using an average of the left and right hemisphere results.

### 3.2. Volume of Cortex Activated at Different Cortical Locations

We derived the volume of cortex that was exposed to at least 75% of the maximum E-field for each of the nine selected cortical locations in the two hemispheres with the TMS intensity set at 150 A/µs and coil oriented towards Nz. The results are shown in Figure 4. The volume of activated cortex was variable and ranged from 3000 to 7000 mm^3^. Unlike the small difference in E-fields at homologous locations, the volume of activated cortex differed substantially between the left and right homologs. For the same E-field input and coil orientation, the volume of BA 3a exposed to ≥75% maximum E-field was 30% larger in the right hemisphere. Activated volumes of BA 41/42, BA 3, and BA 4 differed between 10 and 15% between the two hemispheres. However, E-fields in BA 44, BA 9, and BA 22 in both hemispheres exhibited similar spatial extents. Within the same hemisphere, the activated volumes were found to differ considerably between brain areas. In the left hemisphere, the activated volume was largest in BA 22, while in the right hemisphere, the activated volume was largest in BA 41/42. In both hemispheres, the extent of activation was found to be nearly 50% smaller in the primary motor (BA 4) and sensory areas (BA 2 and 3), despite having the same intensity and orientation parameters as areas in the temporal lobe.

### 3.3. Effect of Varying TMS Intensity on the Maximum E-Field and Its Distribution

**Figure 5:**
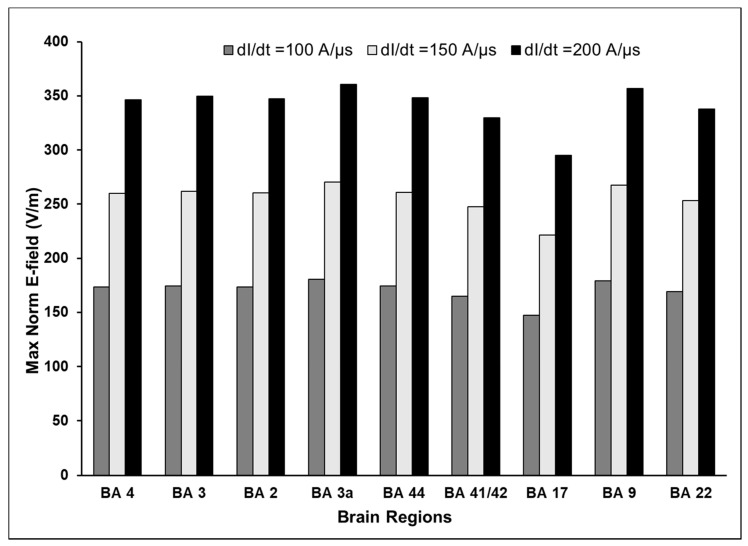
Effect of increasing intensity of the TMS on the induced E-field. The induced E-field is indexed by maximum Norm E field, which increases proportionately with TMS intensity.

Increasing intensity of TMS resulted in a corresponding increase in the maximum E-field in the same manner in all areas examined. The results for coil orientation pointing to the nose are plotted in Figure 5. Across all brain areas examined, the induced E-field on the cortical surface increased by 33% when dI/dt increased by 50 A/µs. We verified that this holds true for the different coil orientations considered in this study. The volume of cortex exposed to ≥75% maximum E-field remained unchanged across the three intensities.

### 3.4. Effect of Varying TMS Coil Orientation on the Maximum E-Field and Its Distribution

**Figure 6:**
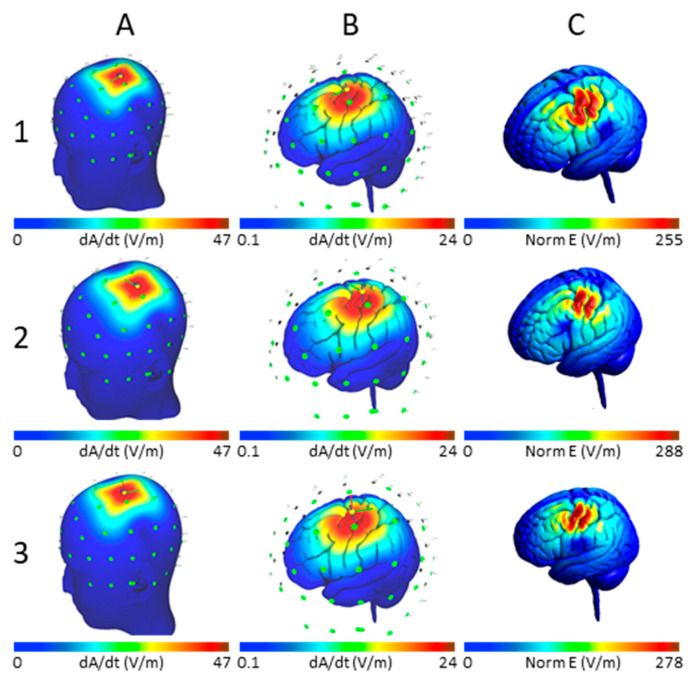
Input and output E-field distributions with TMS coil at position BA 4 and dI/dt = 150 V/m. Coil orientation: Row **1**—Pointing to Nz, Row **2**—Pointing to FCz, Row **3**—Pointing to Oz; E-Field Distribution: Columns: Initial estimation of induced E-field (dA/dt) for the given coil type, position and E-field input superimposed on the scalp (column **A**) and cortical surface (column **B**); Distribution of the final modeled maximum E-field (Norm E-field) on the cortical surface (column **C**). Though the initial estimation of E-field is the same irrespective of coil orientation, the FEM modeled output E-field magnitude and distribution are influenced by the direction of the TMS coil.

**Figure 7:**
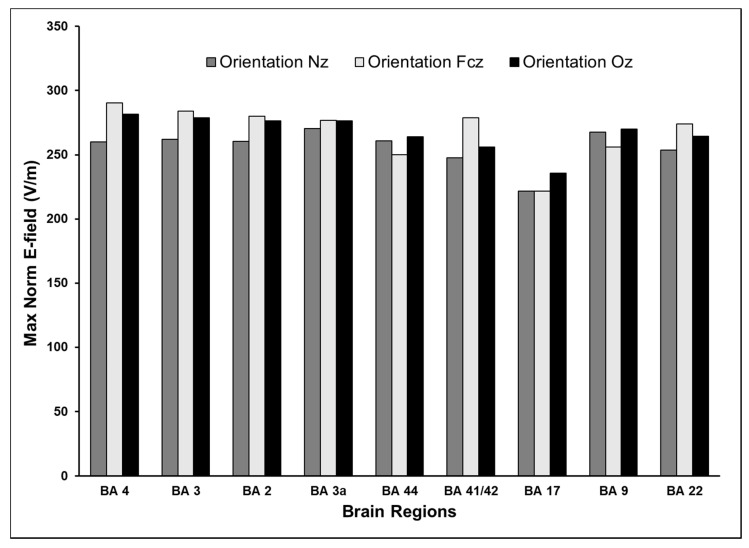
Effect of TMS coil direction on maximum norm E-field. Coil orientations examined were pointing to Nz, FCz, or Oz electrodes on the 10-10 EEG system. The TMS intensity modeled was dI/dt = 150 A/µs. The change in coil orientation had the most impact on the maximum norm E-field for coil at positions BA4 and BA 41/42 while a minimal impact at BA 3a.

We examined the effect of three TMS coil orientations on the induced E-field and its spatial distribution. The E-field distribution changes with the coil orientation in the primary motor cortex in the left hemisphere as shown in Figure 6. The E-fields at different cortical locations for a TMS intensity of dI/dt = 150 A/µs for the three chosen directions (coil pointing to EEG positions: Nz, FCz, and Oz) are plotted in Figure 7. Table 1 summarizes the maximum E-fields estimated for all stimulus intensities and two coil orientations (Nz and FCz) for all brain regions examined.

The standard deviation of the induced E-field for the different coil positions for any given TMS intensity was 5.3% when the coil pointed to the nose (EEG position Nz) and was 7.1% when it pointed to the vertex (EEG position FCz). The greatest percentage differences (>10%) were observed between coil directions in the primary motor cortex (BA 4) and primary auditory cortex (BA 41/42) and the least differences (<3%) were noted in the primary somatosensory cortex (BA 3a) and the primary visual cortex (BA 17), when comparing orientations Nz (pointing to the nose) and FCz (pointing to the vertex). The coil pointing to the vertex was found to be the optimal orientation resulting in highest E-field for primary motor (BA 4) and somatosensory (BA 3 and BA 2) cortices, and primary and secondary auditory cortices (BA 41/42 and BA 22). The standard coil orientation pointing to the nose was not found to be optimal in any brain area.

**Figure 8:**
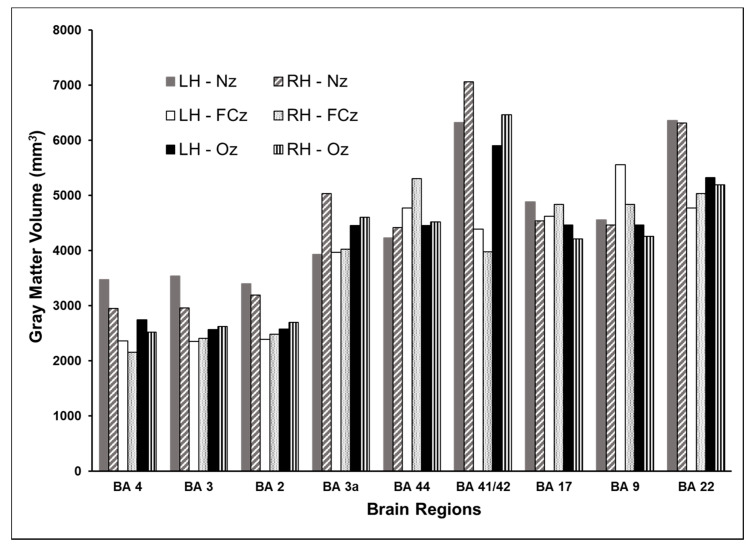
Effect of the TMS coil direction on the volume of activated cortex. Coil orientations examined were pointing to Nz, FCz, or Oz electrodes on 10-10 EEG system. The TMS intensity modeled was dI/dt = 150 A/µs. For most locations, coil pointing to the nose resulted in larger volume of cortex experiencing at least 75% of the maximum E-field than the other two orientations. The coil oriented to the nose also had more variability between the homologous regions in the two hemispheres.

The volume of gray matter activated by different orientations of TMS showed variability between homologous regions in the two hemispheres. Therefore, the data are shown separately for the brain regions in the two hemispheres in Figure 8. For most locations, coil pointing to the nose resulted in a larger volume of cortex experiencing at least 75% of the maximum E-field than the other two orientations considered. The coil oriented to the nose also had more variability between the homologous regions in the two hemispheres. On average, there was 10% (±SD 9%) difference between the activated volumes in the homologous regions. TMS coil pointing to the vertex resulted in smaller volume of cortex exposed to at least 75% of the maximum E-field than when pointing to the nose. There was less variability in the activated volumes between the two hemispheres for the coil oriented to the vertex (7% ± 4% difference) and the occipital lobe (5% ± 3% difference).

## 4. Discussion

We incorporated a 3D FEM of the standard MNI brain in the open-source simulation platform, SimNIBS, to study the effects of varying TMS intensities and coil orientations on the induced E-field and the spatial distribution of activated cortex in nine clinically relevant brain areas. The simulation results of the decay of the induced E-field by the square of the distance of the coil from the scalp agreed well with the measured data (R^2^ = 0.98, Figure 1). This, we believe, is the first reported validation that verifies the accuracy of the modeled Magstim 70 mm figure-of-8 coil in SimNIBS.

First, we found that the volume of activated cortex varied between locations in homologous brain areas (Figure 4). Despite the TMS intensity and orientation being unchanged, there was 10–30% difference in the volume of cortex activated by TMS between certain brain areas. Consistent with previous studies [14,15,16,17,18], we found that for a given TMS intensity and orientation combination, the maximum induced E-field in each of the homologous location pairs was of similar magnitude, with less than 4% difference (Figure 4). While the E-field magnitude data are indicative broadly of symmetry in cortical folding patterns in the homologous areas of the brain in the two hemispheres, the differences in activated volumes point to underlying differences in gray matter architecture. Our results indicate that for some of the brain areas, e.g., dorsolateral prefrontal cortex (BA 9), inferior frontal gyrus (BA 44), and middle temporal gyrus (BA 22), both the magnitude and the extent of activated cortex from one hemisphere can be generalized to the same region in the other hemisphere. However, this does not hold true for other brain areas we examined. Hence, whenever possible, the TMS parameters should be optimized individually even for homologous regions.

Second, the results from this study clearly demonstrate that the induced E-field and the activated volume vary relative to the cortical area being stimulated. The highest E-fields were observed with the coil over the motor cortex and the lowest when placed over the visual cortex for a given coil orientation and TMS intensity, with up to a 27% difference between the motor and visual cortical E-fields. Similarly, the activated volumes were found to differ considerably between brain areas within the same hemisphere. Several areas were found to have notably different volumes of activation, with areas in the frontal lobe being nearly 50% smaller, despite having the same intensity and orientation parameters as areas in the temporal lobe. These results highlight the importance of underlying cortical morphology in the final determination of magnitude of the E-field and the extent of activation observed in a brain area. The findings are of critical relevance in clinical practice where TMS parameters determined at one brain area are used to stimulate a different brain area. For instance, it is standard practice that the intensity and orientation parameters derived at the primary motor cortex are applied at the treatment location of dorsolateral prefrontal cortex in the depression treatment protocol [33,35]. Our results indicate that the TMS parameters derived from the motor cortex very likely underestimate both the magnitude and extent of E-field delivered at the dorsolateral prefrontal cortex. Therefore, future studies should investigate ways to determine and optimize TMS parameters specifically for the brain area being treated or studied.

Third, we studied the effect of changing the intensity of the TMS input on the induced E-field and its extent. Our results show that for any given location and orientation, the induced E-field increases approximately 33% for every 50 A/µs for inputs between 100 and 200 A/µs. This observation is consistent with previous reports from magneto-quasistatic FEM studies [17,31] clinical and animal studies using TMS [37] and the general observation that the induced E-field varies linearly with the intensity of the TMS input. Although, the total volume of cortex stimulated increases in proportion to the TMS intensity, the relative volume of cortex exposed to 75% or more of the maximum field remains the same across all intensities since the spatial distribution of the induced E-field is primarily dependent on the location and orientation of the coil and the conductivity of the underlying tissues and not on the intensity of the input E-field. The findings from this study confirm that the relationship between the E-field in the cortex and TMS intensity is monotonic and can be readily extrapolated. For instance, if the TMS intensity to elicit a behavior is known, for example the motor threshold, then the E-fields for suprathreshold TMS intensities can be accurately estimated.

Fourth, this simulation study found that the coil orientation is a critical TMS parameter that influences the induced E-field and the volume of activated cortex. The induced E-field and volume of cortex varied 10% between different coil orientations. For example, change in coil orientation had the most impact on the maximum E-field at BA 4 and BA 41/42, while no major change was observed at BA 3a. This may reflect that area 3a is located in the deeper layers of the sulcus when compared to other brain areas examined here [38,39]. The volume of gray matter activated by different orientations of TMS showed notable variability between the two hemispheres and between different areas in the same hemisphere. Interestingly, we found that the recommended coil orientation with antero-posterior current flow pointing to the nose [32,33,34] did not achieve the highest E-fields in any of the examined brain areas, but resulted in a larger volume of cortex exposed to at least 75% of the maximum E-field. TMS coil pointing to the vertex often resulted in higher E-fields and a smaller volume of activated cortex. Thus, overall, there was an inverse relationship between the maximum E-field and the activated volume, with smaller volumes of cortex activated at orientations that resulted in higher maximum E-fields. These findings once again suggest that TMS parameters should be optimized individually for the brain area being treated or studied. Such optimization should also consider the trade-off between achieving high E-fields versus stimulating larger volumes of cortex. For example, in functional mapping applications, it is important to achieve more focal E-fields for an accurate localization of eloquent cortex.

Our findings can be explained by the interaction between cortical columns in the underlying cortex and the direction of current flow in the TMS coil. The sensitivity of behavioral effects to coil orientation has been unambiguously demonstrated in the motor system whereby certain orientations are more effective than others at eliciting an evoked response [7,8]. While it is not exactly known how this orientation preference is mediated, there is agreement that TMS primarily stimulates neuronal elements that are aligned parallel to its E-field. However, while the cortical column excitation model hypothesizes that applying E-field parallel to the cortical column is most effective [11], the direct axonal excitation model postulates that TMS chiefly excites axons at terminations or bends in the axon [40,41]. Irrespective of where the TMS interaction occurs, both these models require the E-field to be aligned perpendicular to the sulcus for optimal effects. We have previously found that motor-evoked potential correlated better with induced E-field alignment to the cortical columns than with resting motor threshold [42] and that E-field components were parallel to cortical columns in the TMS activated cortex [43]. More recently, there is empirical evidence that aligning the E-field perpendicular to the sulcus is optimal in the clinical motor [44] and language mapping studies [45,46]. In our simulation study, we believe that for many brain areas, the coil oriented towards the vertex aligned the E-field along the cortical columns better than the orientation pointing to the nose. We propose that by delivering more focal and higher induced E-field, TMS aligned to the cortical column will result in most consistent and reproducible behaviors when compared to those elicited with the standard coil orientation.

While it is important to have a priori knowledge of the most effective orientation for any TMS study, it is especially critical in language mapping since finding the orientation iteratively in real time at each location is almost impossible. Here, computational models can serve as effective predictive tools to determine location/orientation preferences ahead of time. Furthermore, consistent with our findings, other simulation studies in healthy individuals have also shown that the E-field distribution varies from individual to individual based on variability in anatomical details in brain structure [12,46,47]. These findings highlight the need for personalized optimization of cortical location and orientation, over the current norm for stimulation using TMS, which is to use standard, predefined cortical locations and coil orientations for all subjects [48,49].

There are some limitations in our study. For instance, we used isotropic tissue conductivities for the entire brain. Therefore, brains having tissues with different conductivity properties such as tumors and a stroke cannot be accurately modeled. The current model does not differentiate neuronal elements that are known to have a differential response to TMS (e.g., pyramidal neurons and interneurons) and does not account for differing white matter anisotropy across brain regions. Inclusion of these elements will assist in further delineation of the effects of TMS and should be considered in future modeling studies. Another limitation of this study is the requirement of a high resolution MRI image to generate individualized models, which may not be possible and/or cost effective in all individuals. In such instances, existing data from other individualized models can likely be extrapolated to individuals without high resolution MRI, especially if they are from similar demographic and clinical groups. Further, using individualized modeling in a representative sample population can be helpful in identifying specific patient populations and/or neurological conditions where TMS parameters can be generalized or need to be individualized to add value to diagnosis/treatment using TMS. Computational models are facilitating a transition towards personal treatment recommendations for TMS [50].

## 5. Conclusions

Using a 3D, FEM of the standard MNI brain in the open-source simulation platform, SimNIBS, we demonstrated the importance of TMS parameters of intensity and orientation in clinically relevant brain areas. We found that both the maximum induced E-field and the volume of cortex that is activated were strongly influenced by the underlying cortical anatomy and its interaction with TMS coil orientation. Researchers and clinicians should be cognizant that TMS parameters do not readily translate between individuals, hemispheres, and brain regions. Based on our findings, we recommend that whenever possible, the TMS parameters should be optimized individually to each brain area, taking into account both the optimal E-field values and the volume of cortical activation.

The current SimNIBS model used in this study, though simplistic, provides results that agree with clinical results [10] and is a promising tool to study the effect of location and orientation on TMS induced E-field. It provides a basis to incorporate complexities, including neuronal components [8], and tissue segmentation and finer intricacies of the cortical folds that may also affect E-field distributions. In our future studies, we plan to optimize cortical location and coil orientation, given a brain condition and accordingly introduce different tissue and white matter anisotropy, and develop an interface to clinical TMS systems to provide individualized predictions. These advances will pave the way for incorporation of an individualized TMS delivery plan based on patient age, condition, and brain deformities in all clinical and research applications of TMS.

## Figures and Tables

**Table 1:** Simulated induced E-fields for the brain areas in left and right hemispheres with the coil pointing to directions Nz and FCz for different intensities of TMS.

Hemisphere	Brain Region	Brodmann Area	Coordinates (x,y,z)	dI/dt (A/µs)					
				100	150	200	100	150	200
				Max Norm E (V/m)					
				Reference: Nz			Reference: Fcz		
Left	Primary motor cortex	BA 4	(−38,−18.5,54)	170	255	340	192	288	384
Left	Primary somatosensory cortex	BA 3	(−46,−18.5,54)	174	261	348	187	280	374
Left	Primary somatosensory cortex	BA 2	(−48,−20,54)	174	261	349	186	279	372
Left	Primary somatosensory cortex	BA 3a	(−64,−6,14)	182	273	364	184	276	368
Left	Inferior frontal gyrus	BA 44	(−46,16,6)	173	259	346	165	248	331
Left	Primary auditory cortex	BA 41/42	(−60,−22,10)	164	246	328	182	274	365
Left	Primary visual cortex	BA 17	(−8,−96,0)	146	219	292	148	221	295
Left	Dorsolateral prefrontal cortex	BA 9	(−42,36,34)	180	269	359	169	254	338
Left	Middle temporal gyrus	BA 22	(−60,−46,18)	166	249	332	182	273	364
Right	Primary motor cortex	BA 4	(38,−18.5,54)	177	265	353	195	293	390
Right	Primary somatosensory cortex	BA 3	(46,−18.5,54)	175	263	351	192	288	384
Right	Primary somatosensory cortex	BA 2	(48,−20,54)	173	260	346	187	281	374
Right	Primary somatosensory cortex	BA 3a	(64,−6,14)	179	268	357	185	278	371
Right	Inferior frontal gyrus	BA 44	(46,16,6)	176	263	351	168	252	337
Right	Primary auditory cortex	BA 41/42	(60,−18,6)	166	249	332	189	284	378
Right	Primary visual cortex	BA 17	(8,−96,0)	149	224	298	148	222	296
Right	Dorsolateral prefrontal cortex	BA 9	(42,36,34)	178	266	355	172	258	343
Right	Middle temporal gyrus	BA 22	(60,−46,18)	172	258	344	182	275	367

## Abbreviations

BA	Brodmann area
CSF	Cerebrospinal fluid
E-field	Electrical field
EEG	Electroencephalogram
FCz	Electrode position in the 10-10 EEG system at the vertex
FDA	US Food and Drug Administration
FEM	Finite element method
GM	Gray matter
LH	Left hemisphere
MNI	Montreal Neurological Institute
Nz	Electrode position in the 10-10 EEG system at the nose
Oz	Electrode position in the 10-10 EEG system at the occipital pole
RH	Right hemisphere
SimNIBS	Simulation for non-invasive brain stimulation
TMS	Transcranial Magnetic Stimulation
WM	White matter
